# SIRT1-dependent modulation of methylation and acetylation of histone H3 on lysine 9 (H3K9) in the zygotic pronuclei improves porcine embryo development

**DOI:** 10.1186/s40104-017-0214-0

**Published:** 2017-11-01

**Authors:** Katerina Adamkova, Young-Joo Yi, Jaroslav Petr, Tereza Zalmanova, Kristyna Hoskova, Pavla Jelinkova, Jiri Moravec, Milena Kralickova, Miriam Sutovsky, Peter Sutovsky, Jan Nevoral

**Affiliations:** 1 Department of Veterinary Sciences, Faculty of Agriculture, Food and Natural Resources, Czech University of Life Sciences Prague, 6-Suchdol, Prague, Czech Republic; 2 Division of Biotechnology, Safety, Environment and Life Science Institute, College of Environmental and Bioresource Sciences, Chonbuk National University, Iksan, 54596 South Korea; 3 Institute of Animal Science, 10-Uhrineves, Prague, Czech Republic; 4 Proteomic Laboratory, Biomedical Center of Faculty of Medicine in Pilsen, Charles University, Pilsen, Czech Republic; 5 Laboratory of Reproductive Medicine of Biomedical Center, Charles University, Pilsen, Czech Republic; 6 Department of Histology and Embryology, Faculty of Medicine in Pilsen, Charles University, Pilsen, Czech Republic; 7 Division of Animal Science, University of Missouri, Columbia, MO USA; 8 Departments of Obstetrics, Gynecology and Women’s Health, University of Missouri, Columbia, MO USA

**Keywords:** Embryonic development, Epigenetics, H3K9 methylation, SIRT1, Sirtuin

## Abstract

**Background:**

The histone code is an established epigenetic regulator of early embryonic development in mammals. The lysine residue K9 of histone H3 (H3K9) is a prime target of SIRT1, a member of NAD^+^-dependent histone deacetylase family of enzymes targeting both histone and non-histone substrates. At present, little is known about SIRT1-modulation of H3K9 in zygotic pronuclei and its association with the success of preimplantation embryo development. Therefore, we evaluated the effect of SIRT1 activity on H3K9 methylation and acetylation in porcine zygotes and the significance of H3K9 modifications for early embryonic development.

**Results:**

Our results show that SIRT1 activators resveratrol and BML-278 increased H3K9 methylation and suppressed H3K9 acetylation in both the paternal and maternal pronucleus. Inversely, SIRT1 inhibitors nicotinamide and sirtinol suppressed methylation and increased acetylation of pronuclear H3K9. Evaluation of early embryonic development confirmed positive effect of selective SIRT1 activation on blastocyst formation rate (5.2 ± 2.9% versus 32.9 ± 8.1% in vehicle control and BML-278 group, respectively; *P* ≤ 0.05). Stimulation of SIRT1 activity coincided with fluorometric signal intensity of ooplasmic ubiquitin ligase MDM2, a known substrate of SIRT1 and known limiting factor of epigenome remodeling.

**Conclusions:**

We conclude that SIRT1 modulates zygotic histone code, obviously through direct deacetylation and via non-histone targets resulting in increased H3K9me3. These changes in zygotes lead to more successful pre-implantation embryonic development and, indeed, the specific SIRT1 activation due to BML-278 is beneficial for in vitro embryo production and blastocyst achievement.

**Electronic supplementary material:**

The online version of this article (10.1186/s40104-017-0214-0) contains supplementary material, which is available to authorized users.

## Background

Correct formation of maternal and paternal pronuclei in the fertilized mammalian oocyte, the zygote, is required for the first mitotic cell cycle, subsequent zygotic genome activation and successful development of early embryo [1, 2]. Many events, such as protamine-histone replacement [3, 4], protein recycling through ubiquitin-proteasome system (UPS) [5, 6] and correct establishment of euchromatin and heterochromatin [7, 8], lead to genome-wide alterations required for the biogenesis of pronuclei. In addition to these essential genomic and cellular events, pronuclei undergo epigenetic changes, i.e. DNA methylation as well as histone methylation and acetylation, collectively termed the histone code establishment [9–13]. Epigenetic changes in the early zygote include DNA demethylation in both the maternal and paternal pronucleus [14] as well as parent-of-origin specific modifications of pronuclear histone code [9]. However, up-stream factors of histone code in zygote and their influence on embryo development and blastocyst quality are poorly understood.

Sirtuins (SIRTs) are a family of NADP^+^-dependent histone-deacetylases including 7 isoforms with specific subcellular localization patterns [15]. Among them, SIRT1 is the most potent regulator of histone code, present notably in the nucleus and it enhances cell viability by regulating epigenome remodeling [16, 17]. The expression of SIRTs in mammalian oocytes and embryos have been observed [18–22], and the essential role of SIRT1 in oocyte maturation and early embryonic development has been established [19, 23]. Accordingly, beneficial effect of red grape flavonoid resveratrol, a cell protectant/antioxidant substance and a strong activator of SIRT1, on oocyte quality and success of embryonic development is well-known [24–27]; however, we lack the understanding of mechanisms by which SIRT1 enhances oocyte maturation, fertilization and early embryonic development.

Based on somatic cell studies, SIRT1 is able to remove the acetyl group from lysine residues of several histones, resulting in deacetylation of histone H1 on lysine K26 [28, 29], H3 on K9, K14 and K56 [28, 30], and H4 on K8, K12 and K16 [28, 31]. Acetylation of H3K9 is an established marker of translational activity, but it is also frequently associated with DNA damage [32]. Deacetylation of H3K9 makes it available for methyl group addition by histone methyltransferases [33–36]. The involvement of UPS, through the participation of Mouse double minute 2 homolog (MDM2), an E3-type ubiquitin ligase, in SIRT1-mediated H3K9 methylation is indicated [37] and remains the lone consideration of SIRT1 mechanism in the nucleus.

Based on the above knowledge, we hypothesized that SIRT1 affects acetylation-methylation pattern of H3K9 in formatting porcine zygote pronuclei. We also predicted that the SIRT1-modulated H3K9 zygotic histone code establishment will enhance early embryonic development measured by development to blastocyst and blastocyst quality.

## Methods

### Collection and in vitro maturation (IVM) of porcine oocytes

Porcine ovaries were obtained from 6- to 8-month-old non-cycling gilts (a crossbreed of Landrace x Large White) at the local slaughterhouse (Jatky Plzen a.s., Plzen, Czech Republic) and transported to laboratory at 39 °C. Cumulus-oocyte complexes (COCs) were collected from ovarian follicles with a diameter of 2–5 mm by aspiration with a 20-gauge needle and handled in HEPES-buffered Tyrode lactate medium containing 0.01% (*w*/*v*) polyvinyl alcohol (TL-HEPES-PVA). Only fully grown oocytes with evenly dense cytoplasm, surrounded by compact cumuli, were selected for IVM and washed in maturation medium. The medium used for IVM was modified tissue culture medium (mTCM) 199 (Gibco, Life Technologies, UK) supplemented with 0.1% PVA, 3.05 mmol/L D-glucose, 0.91 mmol/L sodium pyruvate, 0.57 L-cysteine, 0.5 μg/mL LH (Sigma-Aldrich, USA), 0.5 μg/mL FSH (Sigma), 10 ng/mL epidermal growth factor (EGF; Sigma), 10% porcine follicular fluid, 75 μg/mL penicillin G and 50 μg/mL streptomycin. After 22 h of culture, the COCs were cultured in TCM199 without LH and FSH for an additional 22 h. The COCs were cultured in 500 μL of the medium covered by mineral oil in a four-well Petri dish (Nunc, Denmark), at 39 °C and 5% CO_2_ in air [38].

### In vitro fertilization (IVF) and culture (IVC) of porcine oocytes and zygotes

After 44 h of IVM, cumulus cells were removed with 0.1% hyaluronidase in TL-HEPES-PVA and the metaphase II (MII) oocytes with extruded first polar body were selected for IVF. The oocytes were washed three-times with modified Tris-buffered medium (mTBM) [38] with 0.2% bovine serum albumin (BSA; A7888; Sigma) and placed into 100 μL drops of mTBM, covered with mineral oil in a 35 mm Petri dish. The dishes were allowed to equilibrate at 38.5 °C and 5% CO_2_ for 30 min before spermatozoa were added for fertilization. Spermatozoa were prepared as follows: 1 mL liquid semen preserved in BTS-based extender was washed twice in phosphate buffered saline (PBS) with 0.1% PVA (PBS-PVA) at 1,500 rpm for 5 min. The last wash was supplemented with MitoTracker CMTRos (400 nmol/L; M7510, Invitrogen) for 10 min at 39 °C, used to tag sperm mitochondria that associate with the paternal pronucleus inside the fertilized oocytes. Labeled spermatozoa were resuspended in mTBM (2.5–5 × 10^7^ spermatozoa per mL) and 1 μL of this sperm suspension was added to the medium containing the oocytes to give a final sperm concentration of 2.5 or 5 × 10^5^ spermatozoa per mL. Oocytes were co-incubated with spermatozoa for 5 to 6 h at 38.5 °C and 5% CO_2_ in air. For zygote acquisition, oocytes were thereafter washed and transferred into 100 μL PZM3 medium [39] containing 0.4% BSA (A6003; Sigma), for further culture for 22 h. Simultaneously, presumed zygotes were cultured in 500 μL of PZM3 medium for 144 h to reach blastocyst stage. PZM3 medium contained different concentrations of SIRT1 activators or inhibitors dissolved in DMSO (in its final concentration 0.1%) as described below. The IVF and IVC studies were repeated three to five times for each treatment regimen.

### Sirtuin activation and inhibition

Activation and inhibition of SIRT1 was performed in PZM3 during IVC of early zygote development. Activators of SIRT1 included resveratrol (3.0, 6.25, 12.5 μmol/L; non-selective sirtuin activator, Abcam, ab120726) and BML-278 (3.0 μmol/L; selective SIRT1 activator, Abcam, ab144536). Inhibitors of SIRT1 included nicotinamide (2.5, 5.0, 7.5 mmol/L; non-selective sirtuin inhibitor, Abcam, ab120864) and sirtinol (10 μmol/L; selective SIRT1 and SIRT2 deacetylase inhibitor, Abcam, ab141263). The effective concentrations of BML-278 (N-Benzyl-3,5-dicarbethoxy-4-phenyl-1,4-dihydropyridine) and sirtinol were chosen based on preliminary experiments conducted to optimize the concentrations of resveratrol and nicotinamide (data not shown). All compounds were dissolved in DMSO, the concentration of which never bypassed 0.5%, as also used for vehicle controls.

### Immunofluorescence and imaging of zygotes

After 22 h of IVC, presumed zygotes were treated with 0.5% pronase for zona pellucida removal and processed as described by Yi et al. [40], with modifications. Briefly, zygotes were fixed in 2% formaldehyde and permeabilized in 0.1% Triton-X-100 in PBS (PBS-TX) for 40 min. Thereafter, zygotes were blocked in 5% normal goat serum (NGS) in PBS-TX for 25 min. A mixture of mouse monoclonal anti-histone H3 tri-methylated K9 antibody (H3K9me3; ab71604, Abcam, UK; 1:200) and rabbit monoclonal anti-histone H3 acetylated K9 antibody (H3K9ac; ab32129, Abcam, Cambridge, UK; 1:200) was applied overnight at 4 °C. Subsequently, the oocytes were washed twice in 1% NGS in BPS-TX before being incubated with fluorescein isothiocyanate (FITC)-conjugated goat anti mouse (GAM) IgG (cat. #62–6511; Invitrogen, Thermo Fisher Scientific Inc., Waltham, MA, USA; 1:200) and cyanine dye (Cy5)-conjugated goat anti-rabbit (GAR) IgG (111–175-144; Jackson ImmunoResearch Laboratories Inc., West Grove, PA, USA), for 40 min at room temperature. Thereafter, the oocytes were washed twice and mounted into slides in a Vectashield medium with 4′6’-diamidino-2-phenylindole (DAPI; Vector Laboratories Inc., Burlingame, CA, USA). SIRT1 was detected by adapting the above protocol for mouse monoclonal antibody clone 1F3 (ab104833, Abcam). Images were acquired using the Ti-U microscope (Nikon Co., Tokyo, Japan) with Clara Interline CCD camera (Andor Technology PLC, Belfast, Northern Ireland) operated by NIS Elements Ar software (Nikon Co., Tokyo, Japan). Negative controls were performed by omitting specific antibodies and these slides were processed at comparable settings. The image analysis was performed by NIS Elements. Signal intensities of pronuclear H3K9ac and H3K9me3 were scaled by a basal signal intensity of corresponding zygote cytoplasm and expressed as a relative acetylation/methylation ratio.

### Evaluation of fertilization and embryonic development

Within the imaging of 22 h zygotes, an assessment of penetration rate, creation of paternal pronucleus/pronuclei and monospermic fertilization was performed. Embryo cleavage was assessed by microscopy at 48 h of IVC; after 144 h of IVC, embryos were fixed in 2% formaldehyde for 40 min at room temperature (RT), washed three times with PBS, permeabilized with PBS-Triton X-100 for 30 min, and stained with 2.5 μg/mL DAPI (DNA staining; Molecular Probes, Eugene, OR, USA) for 40 min. Embryo cleavage, blastocyst formation, and cell number per blastocyst were assessed under an Eclipse Ci fluorescence microscope (Nikon Co., Tokyo, Japan).

### Quantification of fluorescent immunolabeling intensity of the ooplasmic ubiquitin ligase MDM2

Presumed zygotes were subjected to immunofluorescence protocol described above. A mixture of rabbit polyclonal anti-MDM2 antibody (ab137413, Abcam, UK; 1:200) and mouse monoclonal anti-Glyceraldehyde 3-phosphate dehydrogenase antibody (GAPDH; ab8245, Abcam, UK; 1:200) was used. Only zygotes with visible pronuclei were used for further analysis. Images were acquired and analysed as was described. The MDM2 signal intensity was normalized to signal intensity of GAPDH, considered a housekeeping factor with constant expression in pig oocytes [41, 42].

### Western blotting

In vitro matured MII oocytes, cells undergoing to in vitro fertilization assay, and their cumulus cells were used for western blot analysis. Samples were prepared and processed using the method set out by Nevoral et al. [42], with slight modifications. In brief, denuded oocytes and their cumulus cells were precipitated in 80% aceton for at least 60 min and then lysed in 20 μL of Laemmli buffer containing Triton-X-100 (0.003%, v/v) and SDS (0.001%, v/v), enriched with Complete Mini Protease Inhibitor Cocktail (Roche, Switzerland). Samples were boiled and subjected to SDS-PAGE electrophoresis in 12.5% separating gels and blotted using Trans-Blot TurboTM Transfer System (Biorad Laboratories, Steenvoorde, France) onto a PVDF membrane (GE Healthcare Life Sciences, Amersham, UK). After blocking in 5% non-fat milk in TBS with 0.5% Tween-20 (TBS-T) for 60 min at room temperature, the membrane was incubated with mouse monoclonal anti-SIRT1 (ab110304, Abcam, 1:1,000) or rabbit polyclonal anti-MDM2 (ab137413, Abcam, UK; 1:1,000) diluted in TBS-T for 60 min at room temperature. Mouse monoclonal anti-GAPDH loading control antibody (ab8245, Abcam, UK; 1:2,000) was used under same conditions. Subsequently, the membrane was incubated with horseradish peroxidase (HRP)-conjugated goat anti-mouse or anti-rabbit IgG in TBS (1:3,000; Invitrogen, USA) for 60 min at room temperature. Proteins with adequate molecular weight were detected using the ECL Select Western Blotting Detection Reagent (GE Healthcare Life Sciences, Amersham, UK) and visualised by ChemiDocTM MP System (Biorad, France).

### Statistics

Immunofluorescence data are presented as the mean ± S.E.M. of at least 20 zygotes per experimental group. All analysis were performed in at least three independent repetitions. The general linear models (GLM) procedure in SAS package 9.3 (SAS Institute Inc., Cary, NC, USA) was used, followed by Sheffe’s test and Duncan’s multiple range test for zygote analysis and evaluation of early embryonic development, respectively. *P* ≤ 0.05 was considered to be statistically significant.

## Results

### SIRT1 is accumulated in zygotic pronuclei

**Fig. 1:**
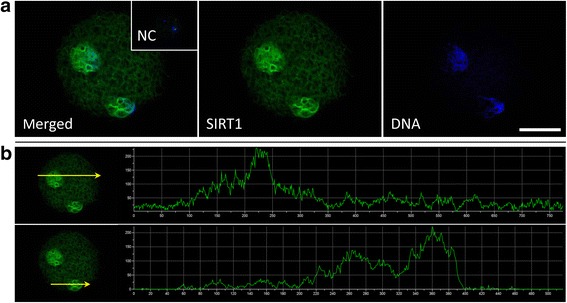
Representative image of SIRT1 immunofluorescence in a 22-h zygote. **a** SIRT1 is exclusively localized in zygote pronuclei. **b** Yellow arrows indicate the level of signal intensity profiles in the respective maternal and paternal pronuclei. NC: negative control where the primary antibody was omitted. Scale bar represents 50 μm

The aim of this experiment was to demonstrate the presence of SIRT1 and verify its subcellular localization in porcine zygotes. Immunocytochemical analysis demonstrated the presence of SIRT1 in both paternal and maternal pronuclei (Fig. 1a) while the intensity of cytoplasmic SIRT1 fluorescence was weak. Such localization was not observed in negative control samples where the primary antibody was omitted. A band of anticipated mass (80.9 kDa; UniProtKB (A7LKB1)) was detected with the same anti-SIRT1 antibody by Western blotting (see Additional file 1).

### Resveratrol increases H3K9 methylation in zygotic pronuclei

**Fig. 2:**
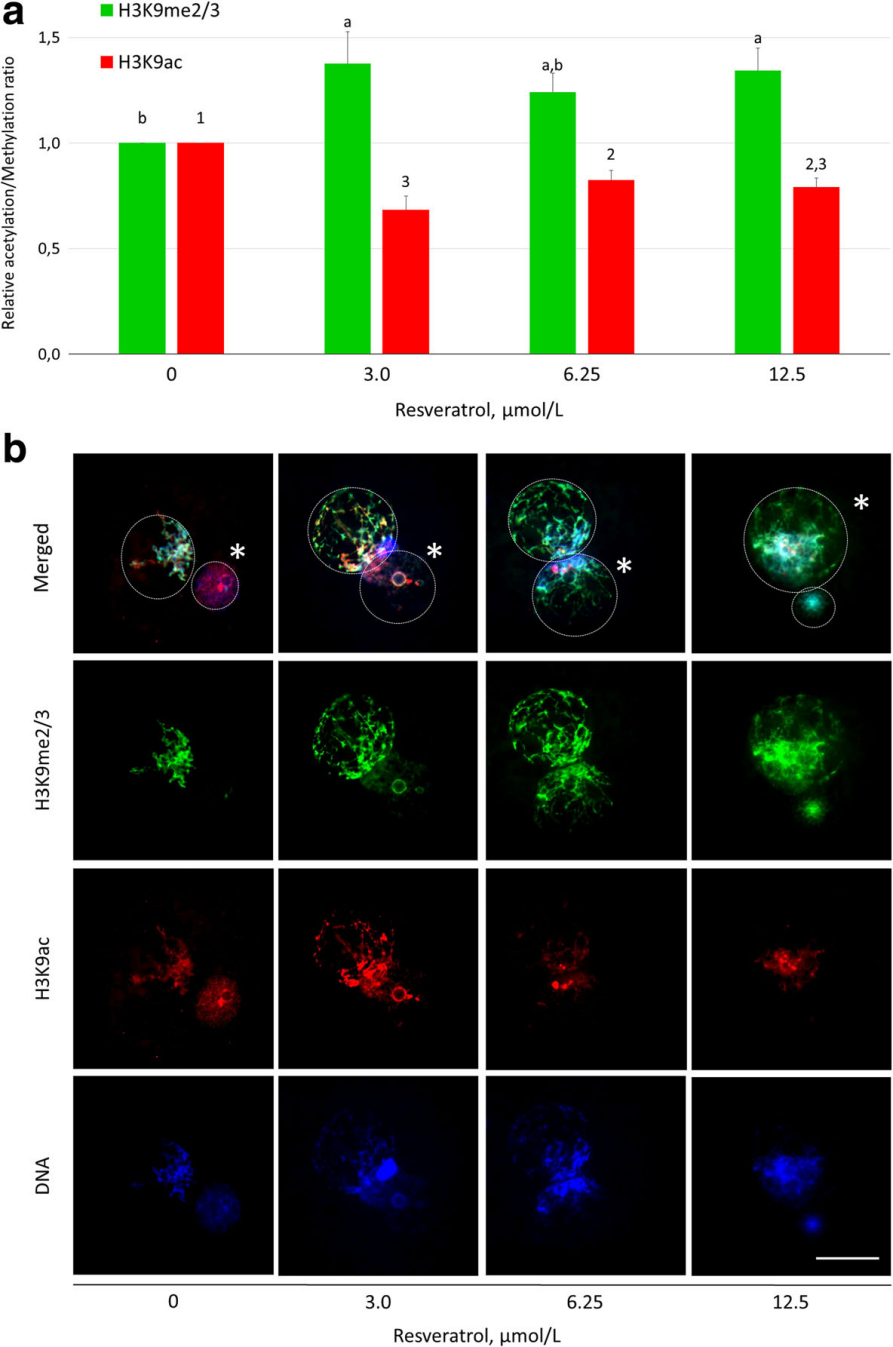
The effect of resveratrol on H3K9 methylation and acetylation in zygotic pronuclei. **a** Fluorescent signal intensities relative to signal intensity of vehicle control (= 1) and **b** representative images of H3K9me3 and H3K9ac in both pronuclei. ^a,b,c;1,2,3^Significant differences in H3K9me3 and H3K9ac, respectively, among experimental groups (*P* ≤ 0.05). Asterisks indicate paternal pronucleus. Scalebar represents 25 μm

To evaluate the effect of SIRT1 activation on pronuclear H3K9 acetylation and methylation, the presumed zygotes were treated by resveratrol, a non-selective activator of SIRTs, and examined by epifluorescence microscopy and relative fluorescence intensity measurement (Fig. 2a, b). Specific antibodies against modified H3K9 modification, validated for zygote imaging by a number of previous studies [43–45], and epifluorescence microscopy followed by image analysis were used. Fertilization rate, paternal pronucleus (PPN) formation and monospermic fertilization rate were simultaneously examined.

A significantly increased intensity of H3K9me3 in both pronuclei was observed in zygotes treated with 3 μmol/L and 12.5 μmol/L resveratrol (1.39 and 1.32 fold, respectively, compared to the vehicle control). Interestingly, the 6.25 μmol/L resveratrol did not show a significant difference from control. Contrary to H3K9 methylation, H3K9 acetylation decreased after resveratrol treatment. Although the H3K9 methylation was affected, resveratrol treatments had no effect on IVF indicators (see Additional file 2).

### Nicotinamide protects pronuclear H3K9 from deacetylation

**Fig. 3:**
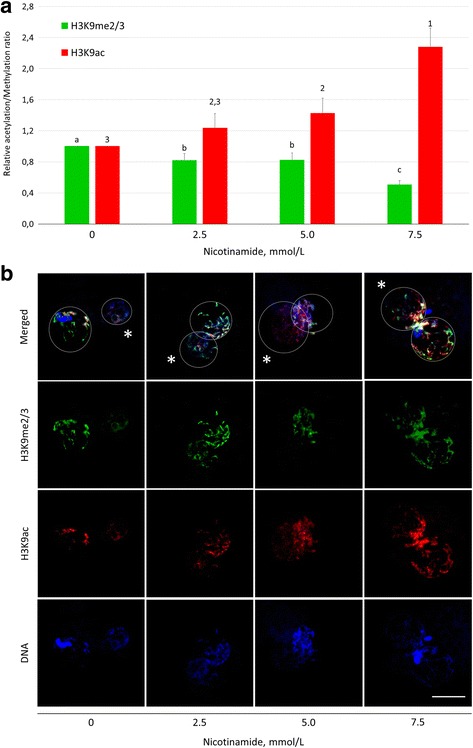
The effect of nicotinamide on H3K9 methylation and acetylation in zygotic pronuclei. **a** Fluorescent signal intensities relative to vehicle control (= 1) and **b** representative images of H3K9me3 and H3K9ac in both pronuclei. ^a,b,c;1,2,3^Significant differences in H3K9me3 and H3K9ac, respectively, among experimental groups (*P* ≤ 0.05). Asterisk indicates paternal pronucleus. Scalebar represents 25 μm

The effect of the inhibition of SIRTs on H3K9 acetylation and methylation in the presumed zygotes treated with nicotinamide (non-selective SIRTs’ inhibitor) was assessed by epifluorescence microscopy and pixel intensity measurement (Fig. 3a, b). In contrast to resveratrol, nicotinamide protected acetylation of H3K9 (1.42 and 2.28-fold increase with 5.0 and 7.5 mmol/L nicotinamide, respectively, compared to vehicle control) and decreased H3K9me3 at 7.5 mmol/L concentration (relative H3K9me3 pixel intensity of 1.00 ± 0.00 versus 0.51 ± 0.05), differences were statistically significant. Similar to resveratrol, nicotinamide did not influence the outcomes of IVF (see Additional file 2).

### Specific modulation of SIRT1 activity amplifies the modification of H3K9

**Fig. 4:**
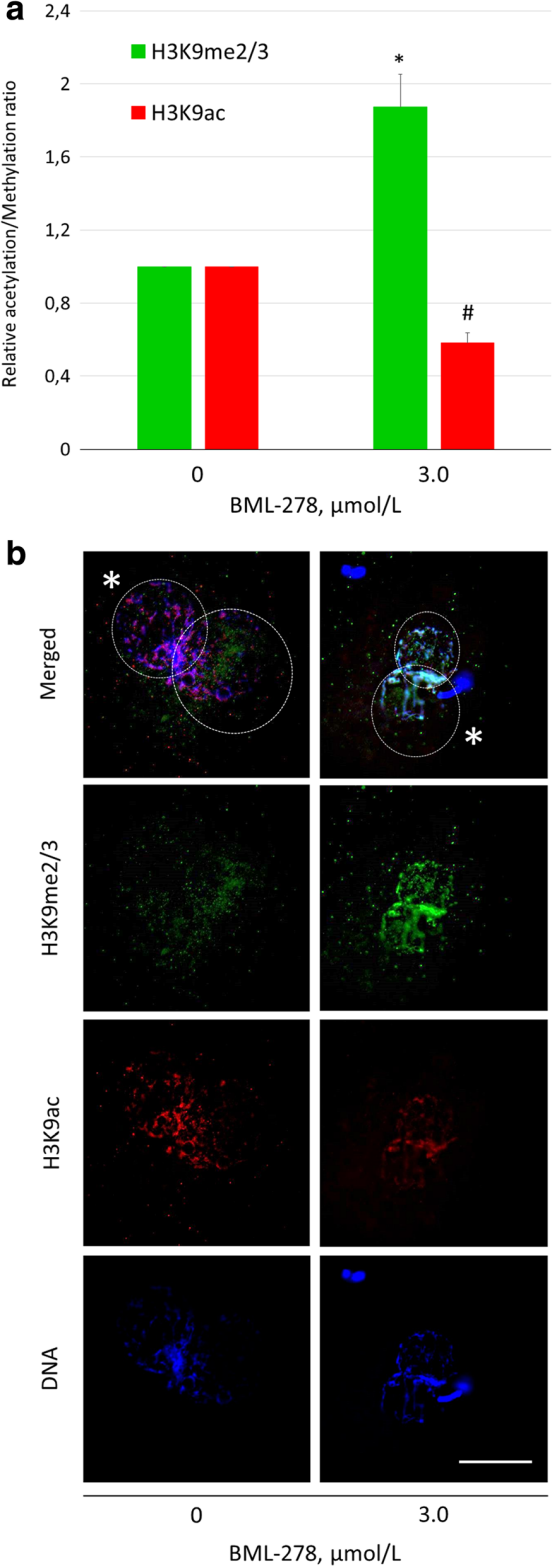
The effect of BML-278 on H3K9 methylation and acetylation in the zygotic pronuclei. **a** Fluorescent signal intensities relative to vehicle control (= 1) and **b** representative images of H3K9me3 and H3K9ac in both pronuclei. *^#^Significant difference between control and treated group (*P* ≤ 0.05). Asterisk indicates paternal pronucleus. Scalebar represents 25 μm

**Fig. 5:**
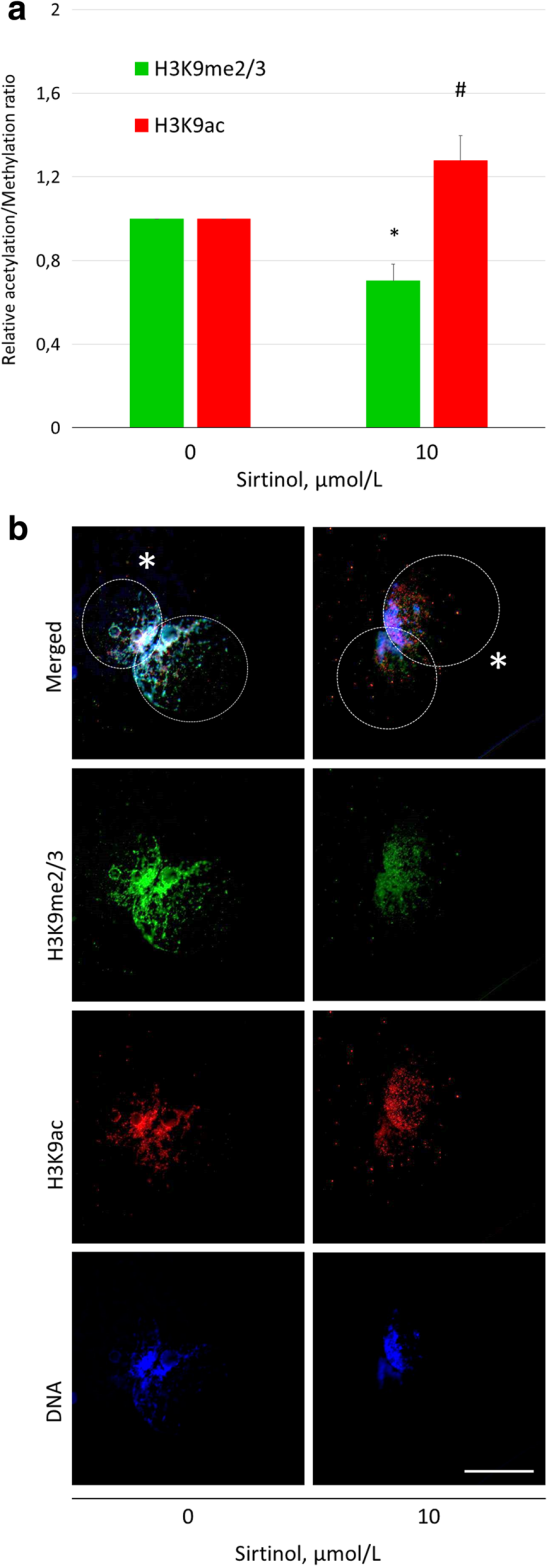
The effect of sirtinol on H3K9 methylation and acetylation in zygotic pronuclei. **a** Fluorescent signal intensities relative to vehicle control (= 1) and **b** representative images of H3K9me3 and H3K9ac in both pronuclei. *^#^Significant differences between control and treated group (*P* ≤ 0.05). Asterisk indicates paternal pronucleus. Scalebar represents 25 μm

Selective SIRT1 activator and inhibitor, BML-278 and sirtinol, respectively, were used for modulation of H3K9 methylation and acetylation in the treated and control zygotes (Figs. 4 and 5). Treatment with BML-278 increased the H3K9me3 and reciprocally decreased H3K9ac (change of 1.87 ± 0.18-fold and 0.58 ± 0.05-fold in H3K9me3 and H3K9ac, respectively, compared with control; *P* ≤ 0.05), (Fig. 4). In reverse, sirtinol significantly decreased H3K9me3 and increased H3K9ac to 0.70 ± 0.08 and 1.28 ± 0.12-fold change over control, respectively (Fig. 5). Similar to resveratrol and nicotinamide, the fertilization and monospermy/polyspermy rates were not affected (see Additional file 2).

### SIRT1-modulation of H3K9 at the zygote stage improves subsequent embryonic development

To assess the effect of SIRT1-modulating treatments on preimplantation embryo development and blastocyst quality, fertilized oocytes were cultured in PZM3 medium with varying concentrations of SIRT1 activator or inhibitor for 144 h.

A significantly lower percentage (39.2 ± 5.2%) of cleaved zygotes was observed in embryos cultured with 12.5 μmol/L resveratrol compared to vehicle control treatment (65.9 ± 8.3%; *P* ≤ 0.05) or to other activator/inhibitor treatments (Table 1). The inhibition of SIRT1 by sirtinol did not impact blastocyst formation rate. However, the rate of blastocyst formation was significantly increased when embryos were cultured in the presence of 3 μmol/L BML-278 (32.9 ± 8.1; *P* ≤ 0.05). There were no significant differences in average cell number per blastocyst among treatment groups (Additional file 3). Representative blastocyst of each treated group is shown in Additional file 3.

**Table 1:** Embryonic development and blastocyst formation after 144 h IVC with SIRT1 activators or inhibitors

		No. of fertilized oocytes	No. of cleaved oocytes (mean % ± SEM)	No. of blastocysts (mean % ± SEM)	Mean cell No. per blastocyst (mean ± SEM)
DMSO, % (v/v)	0.5	38	28 (65.9 ± 8.3)^a^	2 (5.2 ± 2.9)^bc^	36.0 ± 5.0^a^
Resveratrol, μmol/L	3	67	47 (67.7 ± 10.6)^a^	4 (6.2 ± 2.8)^bc^	39.3 ± 11.3^a^
	6.25	51	36 (69.9 ± 6.4)^a^	0 (0.0 ± 0.0)^c^	–
	12.5	50	20 (39.2 ± 5.2)^b^	0 (0.0 ± 0.0)^c^	–
Nicotinamide, mmol/L	2.5	50	39 (76.7 ± 6.9)^a^	7 (16.7 ± 8.8)^abc^	30.9 ± 4.2^a^
	5	70	45 (64.1 ± 1.4)^a^	7 (9.7 ± 6.2)^bc^	36.7 ± 4.5^a^
	7.5	69	45 (66.7 ± 4.6)^a^	13 (21.4 ± 8.4)^ab^	34.9 ± 3.1^a^
BML-278, μmol/L	3	68	44 (62.7 ± 7.1)^a^	18 (32.9 ± 8.1)^a^	38.4 ± 4.2^a^
Sirtinol, μmol/L	10	69	44 (63.5 ± 4.6)^a^	4 (6.5 ± 3.7)^bc^	23.5 ± 1.3^a^

^a,b,c^Different superscripts within the same column were significantly different at *P* ≤ 0.05

### Specific activation of SIRT1 reduces the fluorescent immunolabeling intensity of the ooplasmic ubiquitin ligase MDM2

**Fig. 6:**
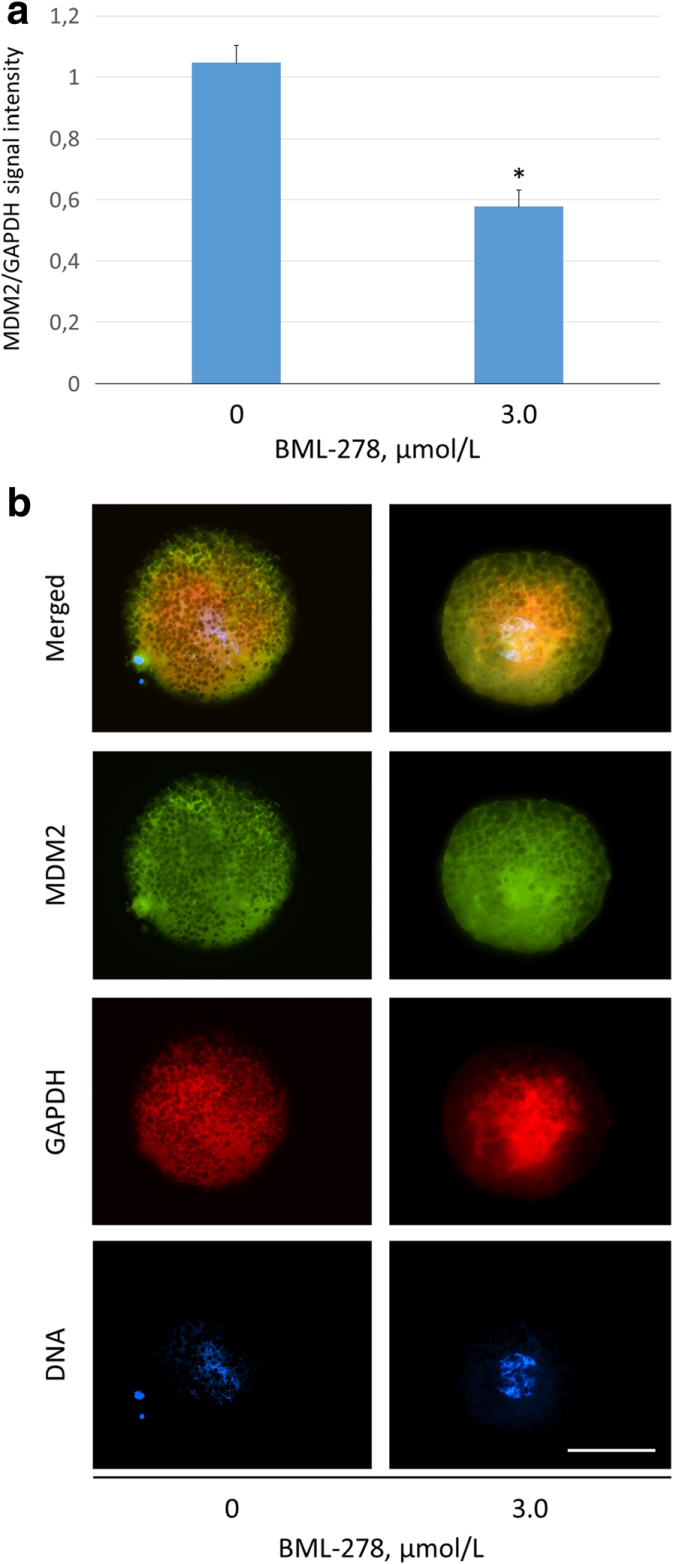
Quantification of MDM2 signal intensity in 22 h zygotes after treatment with BML-278. **a** Fluorescent signal intensity of MDM2 was normalized against housekeeping protein GAPDH. **b** Representative images show MDM2 and GADPH in control and treated zygotes. *Significant difference between control and treated group (*P* ≤ 0.05). Scalebar represents 50 μm

Based on published evidence of SIRT1 mediating the regulation of MDM2 and vice versa [37, 46], we detected MDM2 in matured MII oocytes due to western blotting (see Additional file 1) and measured the fluorescent signal intensity of in situ immunolabeled MDM2 in the zygotes cultured for 22 h under control and SIRT1 stimulating conditions. The signal intensity was normalized to GAPDH. A significant reduction of GAPDH-normalized MDM2 signal intensity (1.13 ± 7.4 versus 0.62 ± 7.7 in vehicle control and treated zygotes; *P* ≤ 0.05) was observed after SIRT1 activation with BML-278 (3 μmol/L) (Fig. 6), indicating significant decrease of MDM2 protein amount.

## Discussion

Histone deacetylase SIRT1 has been shown to deacetylate a number of histone protein Lys-residues including but not limited to H1K26 [28, 29], H3 K9, H3K14 and H3K56 [28, 30], and H4K8, H4K12 and H4K16 [28, 31]. Moreover, SIRT1 activity leads to methylation of H3K9, and this lysine residuum represents a dual target of SIRT1 through both direct histone targeting and indirect non-histone targeting of other enzymes involved in the modifications of H3K9 (reviewed in [47]). Our study is the first to show that the SIRT1 accumulates in the pronuclei of a mammalian zygote and favors methylation of pronuclear H3K9. Our observations are in accordance with predominant SIRT1 presence in cell nucleus and an overall positive effect of SIRT1 activity on cell lifespan, accompanied with histone methylation, as described [28, 31]. Furthermore, we increased the percentage of blastocyst formation by embryo treatment with a specific, SIRT1 activating synthetic compound, BML-278 [48]. This observation agrees with previously documented beneficial effect of resveratrol, a naturally occurring general sirtuin activator, on preimplantation embryo development [24–26]. Our study thus solidifies previously anecdotal evidence of SIRT1 involvement in the establishment and remodeling of zygotic epigenome. In particular, we show that pharmacological modulation of SIRT1 alters pronuclear histone code in a manner affecting the developmental potential of the preimplantation embryo.

Treatment of porcine zygotes with resveratrol, a non-selective, SIRT-family wide activator increased H3K9me3 methylation after 22 h of IVC. Inversely, such treatment decreased H3K9 acetylation supporting the assumption that histone methylation and acetylation replace each other [49, 50]. Indeed, nicotinamide, a non-selective sirtuin inhibitor, had an opposite effect on pronuclear H3K9 modification, wherein H3K9me3 was decreased and H3K9ac was protected in the treated zygotes. While the involvement of SIRT1 in aforementioned modifications of pronuclear H3K9 is plausible, such findings are based on wide-spectrum activation or inhibition of SIRT family, while other effects should be considered as well, such as the antioxidant/reactive oxygen-scavenger action of resveratrol [51]. Hence, BML-278 and sirtinol, the selective SIRT1 activator and inhibitor, respectively, were used. Favoring SIRT1 over SIRT2 or SIRT3 as a substrate [48], BML-278 treatment lead to higher H3K9 methylation and reduced H3K9 acetylation; sirtinol had an opposite, complementary effect. These observations agree with previous studies on sirtinol [19]. Compared to naturally occurring compounds, synthetic small molecules such as BML-278 and sirtinol have a more specific and more prominent effect on SIRT1 activity without secondary targets. Notably, BML-278 can be used for pharmacological modulation of SIRT1 signaling. On the other hand, molecular mechanism of BML-278 remains unknown and there are few alternative SIRT1 activators with suggested resveratrol-like allosteric mechanism mediating interactions between SIRT1 and its substrates [52, 53]. Activating effect of BML-278 is substrate-selective at a level similar to resveratrol [54, 55].

Early zygote features partially demethylated chromatin, resulting from pronuclear histone asymmetry [56, 57] and ongoing, active DNA demethylation [58–60, 14]. Based on the differential tagging of pronuclei with MitoTracker, we found the hyperacetylation of paternal pronucleus on H3K9 and higher H3K9me3 in maternal pronucleus, but no-detectable SIRT1 effect on the asymmetry of pronuclear histone code. The pattern of post-translationally modified histones in pronuclei mirrors DNA methylation status and points out the positive correlation of DNA- and histone H3K9 methylation, indicated earlier [61, 62]. Accordingly, DNA methyltransferase DNMT1 is regulated by H3K9me3-heterochromatin protein 1α complex and crosstalk between DNMTs and histone methyltransferases is obvious [63–65]. Although histone acetylation in general accompanies gene expression and in some cases causes DNA fragmentation [66], H3K9me3 is specifically associated with gene silencing and heterochromatin establishment [67, 68]. These changes are beneficial for chromatin stability and cell longevity; however, they suppress gene expression [67, 69, 70]. With respect to major zygotic genome activation (MZGA) when gene transcriptional activity is desired, SIRT1-derived histone methylation and gene silencing seems to be strictly selective in not affecting promoters and genes of which the expression is essential for early embryonic development.

In addition to histone targets of SIRT1, non-histone substrates may be involved in SIRT1 action in the zygote [71, 72]. Suppressor of variegation 3–9 homolog 1 (SUV39H1) is one of non-histone targets of SIRT1, responsible for SIRT1-mediated increase of H3K9me3 [33, 35]. SIRT1-regulated modification of SUV39H1 activity occurs by two mechanisms: SUV39H1 deacetylation on K266 [35] and indirect protection of SUV39H1 protein via suppression of its polyubiquitination by Mouse double minute 2 homolog (MDM2), which is an E3-type ubiquitin ligase responsible for proteasomal degradation of SUV39H1 [37]. The second mechanism points to a crosstalk of SIRT1 and UPS [73, 74] during SIRT1-modulation of the histone code. However, current knowledge of SIRT1 involvement in ubiquitin ligation and UPS-mediated proteolysis remains incomplete. Therefore, we performed the MDM2 fluorescent signal intensity measurement after BML-278 treatment. Our findings of decreased MDM2 signal intensity support the role of SIRT1 in MDM2-SUV39H1-H3K9me3 signaling, indicated earlier [37]. Based on such evidence, we can consider SIRT1 as a negative regulator of MDM2, presumably affecting MDM2 via lysine deacetylation and thus exposure of lysine residues for ubiquitination-mediated autocatalytic loop [37, 75, 76]. Such a scenario agrees with observation of SIRT-promoted protein degradation of β-TrPC E3 ligase [77].

Our study thus provides the partial understanding of how resveratrol improves embryonic development in vitro via SIRT1 activity modulation. The effect of resveratrol was compared with sirtuin inhibitors and BML-278, a specific SIRT1 activator. Surprisingly, resveratrol showed no positive effect on development to blastocyst, while nicotinamide, a non-selective sirtuin family inhibitor actually increased the blastocyst formation rate. These results indicate non-specific effects of nicotinamide and are in agreement with the observations of improved embryonic development after treatment with trichostatine, an inhibitor of histone deacetylases [78–80]. Compared to resveratrol, BML-278, a specific SIRT1 activator, significantly improved early embryonic development. The wide scale modification of histone code favoring methylation of specific loci is a plausible epigenetic mechanism of SIRT1 action on early embryonic development. Moreover, SIRT1 factors in the sperm-derived transgenerational inheritance [81] and further experiments focused on specific SIRT1-affected loci during embryogenesis and SIRT1-modulated epigenetic memory are needed.

Improving blastocyst formation rate through SIRT1 stimulation could be applied to optimization of assisted reproductive technologies such as somatic cell nuclear transfer (SCNT) in animals and assisted fertilization in human infertility patients, wherein the maximizing of embryo developmental competence is paramount to the success of subsequent embryo transfer. Based on our observation, the adequate modulation of SIRT1 in the in vitro fertilized oocytes and embryos could also benefit the production of genetically modified pigs for biomedical research and production agriculture [82, 83]. This work thus offers a new approach to increasing the efficiency of reproductive biotechnologies for creation of biomedical animal models as well as for human assisted reproduction.

Further experiments will be needed to more precisely understand molecular mechanisms, including SIRT1 signal pathways, leading to successful pre-implanted embryonic development.

## Conclusions

Specific activation of SIRT1 via BML-278 modulated zygotic histone code, expressed by methylated and acetylated H3K9 in zygote pronuclei. In addition to increased pronucleic H3K9me3, activity of MDM2, a non-histone target of SIRT1, has been described after BML-278 treatment. The positive effect of 3.0 μmol/L BML-278 on blastocyst achievement is obvious and, assumably, is a result of described changes on histone code and non-histone targets in one-cell zygote. BML-278 as a novel compound and its molecular action through SIRT1 signalization in porcine embryo represents a promising tool utilizable in biotechnology and assisted reproduction of animals and human.

## Additional file

Additional file 1: Detection of SIRT1 (A) and MDM2 (B) in matured MII oocytes and their cumulus cells. (DOCX 107 kb)
Additional file 2: Results of IVF after 22 h of IVC with SIRT1 activators and inhibitors. (DOCX 19 kb)
Additional file 3: Embryonic development and blastocyst formation after treatment with SIRT1 activators and inhibitors. (DOCX 163 kb)

## Abbreviations

DNMT: DNA methyltransferase
EGF: Epidermal growth factor
FSH: Follicle stimulating hormone
GAPDH: Glyceraldehyde-3-phosphate dehydrogenase
H3K9ac: Acetylation of histone H3 on lysine K9
H3K9me3: Trimethylation of histone H3 on lysine K9
IVC: In vitro culture of embryos
IVF: In vitro fertilization
LH: Luteinizing hormone
MDM2: Mouse double minute 2 homolog, E3-type ubiquitin ligase
MII: Metaphase II
mTBM: Modified Tris-buffered medium
mTCM: Modified tissue culture medium
MZGA: Major zygotic genome activation
PPN: Paternal pronucleus
PZM3: Porcine zygote medium
SCNT: Somatic cell nuclear transfer
SIRT1: Silent mating type information regulator 2 homolog 1, NADP + −dependent histone-deacetylase
SUV39H1: Suppressor of variegation 3–9 homolog 1, histone –lysine N-methyltransferase
TL-HEPES-PVA: HEPES-buffered Tyrode lactate medium with polyvinyl alcohol
UPS: Ubiquitin-proteasome system
β-TrPC E3 ligase: β-Transducin repeat-containing protein
