# Supplementary material for: SIRT1-dependent modulation of methylation and acetylation of histone H3 on lysine 9 (H3K9) in the zygotic pronuclei improves porcine embryo development
Source: J Anim Sci Biotechnol. 2017 Nov 1;8:83. doi: 10.1186/s40104-017-0214-0 (PMC5664433; doi:10.1186/s40104-017-0214-0)
Supplement: Additional file 1: — Detection of SIRT1 (A) and MDM2 (B) in matured MII oocytes and their cumulus cells. (DOCX 107 kb) [file 40104_2017_214_MOESM1_ESM.docx]

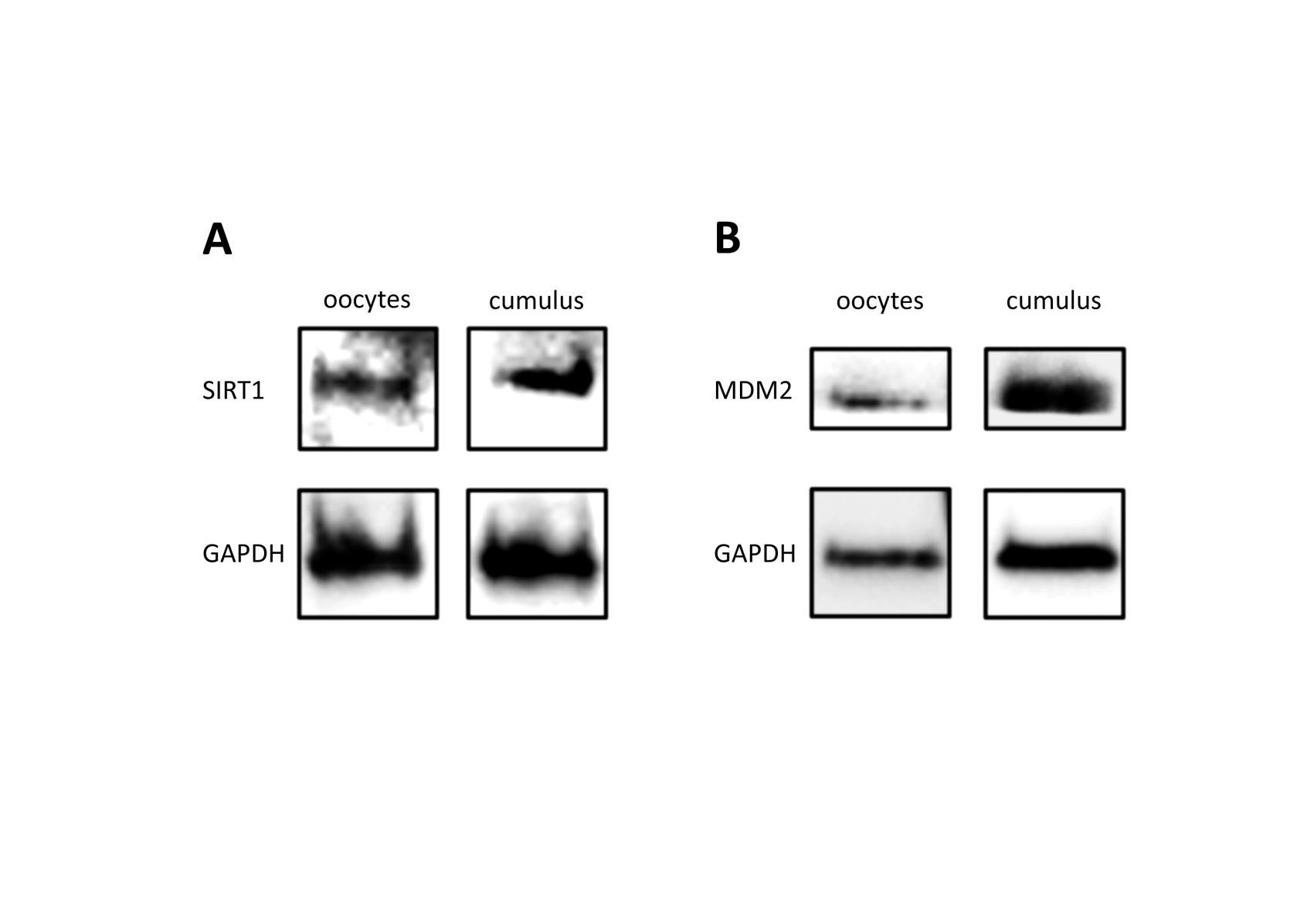


**Fig. 1.1** Detection of SIRT1 (A) and MDM2 (B) in matured MII oocytes and their cumulus cells. The specifity of used antibodies was verified through the detection in anticipated mass 80.9 kDa and 55.4 kDa for SIRT1 (UniProtKB (A7LKB1)) and MDM2 (UniProtKB (A7Y496)), respectively
