# Supplementary material for: SIRT1-dependent modulation of methylation and acetylation of histone H3 on lysine 9 (H3K9) in the zygotic pronuclei improves porcine embryo development
Source: J Anim Sci Biotechnol. 2017 Nov 1;8:83. doi: 10.1186/s40104-017-0214-0 (PMC5664433; doi:10.1186/s40104-017-0214-0)
Supplement: Additional file 2: — Results of IVF after 22 h of IVC with SIRT1 activators and inhibitors. (DOCX 19 kb) [file 40104_2017_214_MOESM2_ESM.docx]

**Table 2.1** Results of IVF after 22hr of IVC with resveratrol

|  | | No. of fertilized oocytes | No. of penetrated oocytes (mean % ± SEM) | No. of oocytes with PPN (mean % ± SEM) | No. of monospermic fertilization  (mean % ± SEM) |
| --- | --- | --- | --- | --- | --- |
| DMSO, % (v/v) | 0.5 | 119 | 71 (59.7±13.9)^a^ | 66 (93.0±3.4)^a^ | 43 (60.6±11.4)^a^ |
| Resveratrol, μmol/L | 3 | 81 | 32 (39.5±8.0)^a^ | 30 (93.8±4.1)^a^ | 14 (43.8±13.4)^a^ |
|  | 6.25 | 100 | 62 (62.0±6.4)^a^ | 48 (77.4±7.1)^a^ | 38 (61.3±8.3)^a^ |
|  | 12.5 | 112 | 64 (57.1±10.8)^a^ | 47 (73.4±6.2)^a^ | 45 (70.3±9.2)^a^ |

PPN: paternal pronucleus (pronuclei). No. of oocytes with PPN and monospermic fertilization were calculated from penetrated oocytes. ^a,b,c^Different superscripts withinthe same column were significantly different at P<0.05.

**Table 2.2** Results of IVF after 22hr IVC with nicotinamide

|  | | No. of fertilized oocytes | No. of penetrated oocytes (mean % ± SEM) | No. of oocytes with PPN (mean % ± SEM) | No. of monospermic fertilization  (mean % ± SEM) |
| --- | --- | --- | --- | --- | --- |
| DMSO, % (v/v) | 0.5 | 203 | 111 (54.7±8.1)^a^ | 89 (80.2±5.4)^a^ | 45 (40.5±8.3)^a^ |
| Nicotinamide, mmol/L | 2.5 | 57 | 48 (84.2±11.2)^a^ | 45 (93.8±6.7)^a^ | 14 (29.2±11.3)^a^ |
|  | 5.0 | 101 | 77 (76.2±9.6)^a^ | 67 (87.0±5.1)^a^ | 36 (46.8±7.5)^a^ |
|  | 7.5 | 50 | 27 (54.0±1.6)^a^ | 22 (81.5±9.1)^a^ | 22 (81.5±5.1)^a^ |

PPN: paternal pronucleus (pronuclei). No. of oocytes with PPN and monospermic fertilization were calculated from penetrated oocytes. ^a,b,c^Different superscripts within the same column were significantly different at P<0.05.

**Table 2.3** Results of IVF after 22hr IVC with BML-278

|  | | No. of fertilized oocytes | No. of penetrated oocytes (mean % ± SEM) | No. of oocyte with PPN (mean % ± SEM) | No. of monospermic fertilization  (mean % ± SEM) |
| --- | --- | --- | --- | --- | --- |
| DMSO, % (v/v) | 0.5 | 195 | 79 (40.5±9.4)^a^ | 50 (63.3±12.6)^a^ | 42 (53.2±12.5)^a^ |
| BML-278, μmol/L | 3.0 | 215 | 92 (42.8±9.5)^a^ | 76 (82.6±6.7)^a^ | 39 (42.4±10.4)^a^ |

PPN: paternal pronucleus (pronuclei). No. of oocytes with PPN and monospermic fertilization were calculated from penetrated oocytes. ^a,b,c^Different superscripts within the same column were significantly different at P<0.05.

**Table 2.4** Results of IVF after 22hr IVC with sirtinol

|  | | No. of fertilized oocytes | No. of penetrated oocytes (mean % ± SEM) | No. of oocytes with PPN (mean % ± SEM) | No. of monospermic fertilization  (mean % ± SEM) |
| --- | --- | --- | --- | --- | --- |
| DMSO, % (v/v) | 0.5 | 170 | 71 (41.8±7.6)^a^ | 54 (76.1±11.2)^a^ | 36 (50.7±10.9)^a^ |
| Sirtinol, μmol/L | 10 | 168 | 71 (42.3±8.7)^a^ | 61 (85.9±5.7)^a^ | 31 (43.7±6.7)^a^ |

PPN: paternal pronucleus (pronuclei). No. of oocytes with PPN and monospermic fertilization were calculated from penetrated oocytes. ^a,b,c^Different superscripts within the same column were significantly different at P<0.05.
