# Supplementary material for: SIRT1-dependent modulation of methylation and acetylation of histone H3 on lysine 9 (H3K9) in the zygotic pronuclei improves porcine embryo development
Source: J Anim Sci Biotechnol. 2017 Nov 1;8:83. doi: 10.1186/s40104-017-0214-0 (PMC5664433; doi:10.1186/s40104-017-0214-0)
Supplement: Additional file 3: — Embryonic development and blastocyst formation after treatment with SIRT1 activators and inhibitors. (DOCX 163 kb) [file 40104_2017_214_MOESM3_ESM.docx]

**Table 3.1** Embryonic development and blastocyst formation rates after IVC with different concentrations of vehicle (DMSO)

|  | | No. of fertilized oocytes | No. of cleaved oocytes (mean % ± SEM) | No. of blastocysts (mean % ± SEM) | Mean cell No. per blastocyst  ± SEM |
| --- | --- | --- | --- | --- | --- |
| DMSO, % (v/v) | 0 | 71 | 46 (63.7±3.8)^a^ | 10 (14.0±2.6)^a,b^ | 41.8±7.5^a^ |
|  | 0.1 | 30 | 19 (63.3±3.3)^a^ | 0 (0.0±0.0)^b^ | - |
|  | 0.5 | 38 | 28 (65.9±8.3)^a^ | 2 (5.2±2.9)^a,b^ | 36.0±5.0^a^ |
|  | 1.0 | 40 | 30 (76.2±11.3)^a^ | 0 (0.0±0.0)^b^ | - |

^a,b,c^Different superscripts within in the same column mark significant difference at P<0.05.

**Table 3.2** Embryonic development and blastocyst formation rates after 144hr IVC with specific SIRT1 activator (BML-278) or inhibitor (sirtinol)

|  | | No. of fertilized oocytes | No. of cleaved oocytes (mean % ± SEM) | No. of blastocyst (mean % ± SEM) | Mean cell No. per blastocyst ± SEM |
| --- | --- | --- | --- | --- | --- |
| DMSO, % (v/v) | 0.5 | 38 | 28 (65.9±8.3)^a^ | 2 (5.2±2.9)^bc^ | 36.0±5.0^a^ |
| BML-278, μmol/L | 1.5 | 67 | 47 (70.2±7.3)^a^ | 10 (14.5±7.2)^bc^ | 32.7±2.5^a^ |
|  | 3.0 | 68 | 44 (62.7±7.1)^a^ | 18 (32.9±8.1)^a^ | 38.4±4.2^a^ |
| Sirtinol, μmol/L | 1.0 | 72 | 52 (74.7±5.7)^a^ | 12 (15.3±4.5)^bc^ | 32.9±4.7^a^ |
|  | 5.0 | 68 | 48 (71.5±9.2)^a^ | 5 (7.1±2.1)^bc^ | 31.0±4.6^a^ |
|  | 10 | 69 | 44 (63.5±4.6)^a^ | 4 (6.5±3.7)^bc^ | 23.5±1.3^a^ |

^a,b,c^Different superscripts within in the same column mark significant difference at P<0.05

**
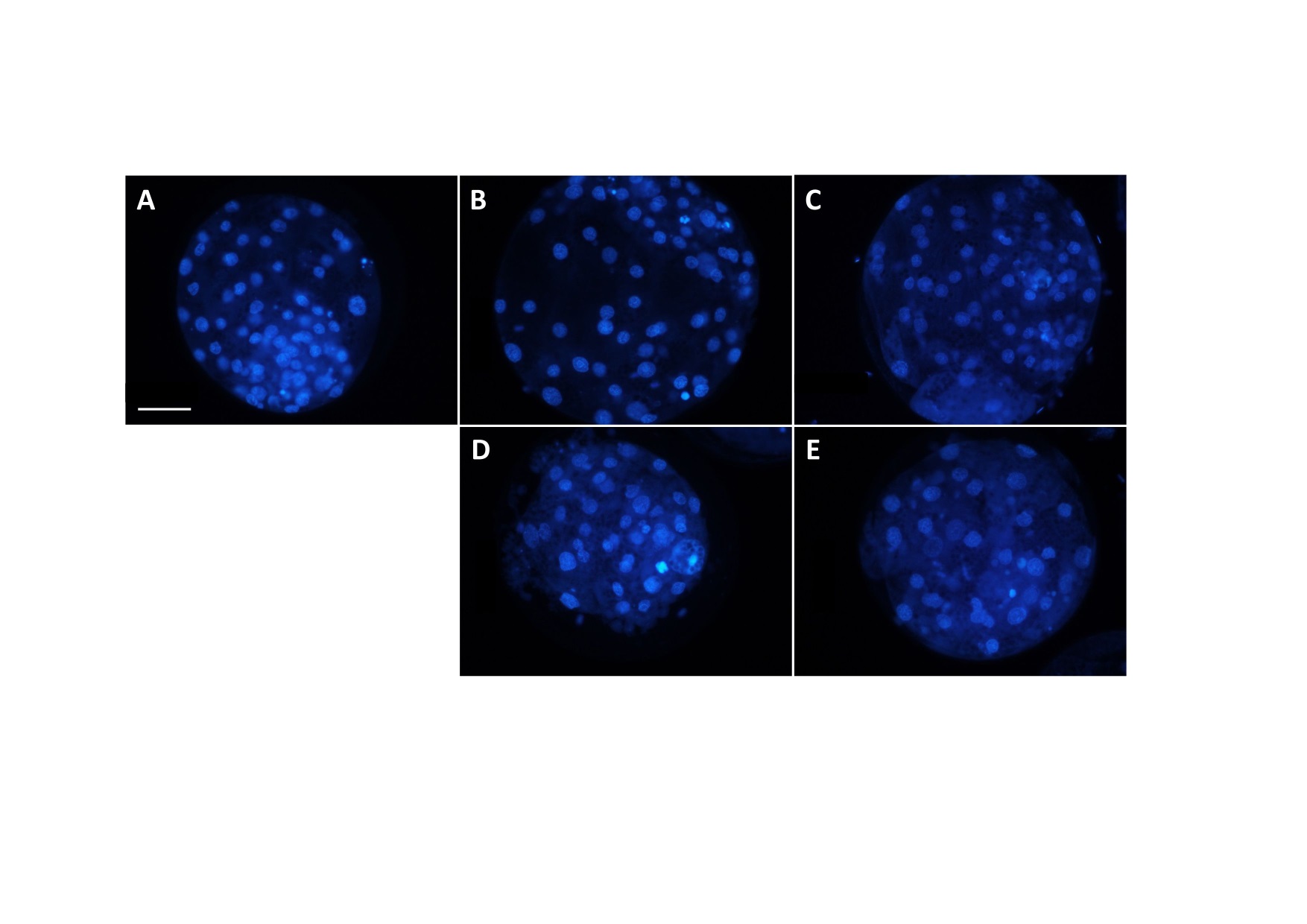
**

**Fig. 3.1** Representative images of blastocysts after 144hr IVC (A), and treatment with 3.0 μmol/L resveratrol (B), 3.0 μmol/L BML-278 (C), 7.5 mmol/L nicotinamide (D) or 10 μmol/L sirtinol (E). The scalebar represents 50 μm
